# Anticoagulant-free preparation of autologous platelet-rich plasma (PRP) / fluid platelet-rich fibrin (f-PRF): a pre-clinical comparative performance study

**DOI:** 10.3389/fphar.2026.1785884

**Published:** 2026-03-04

**Authors:** Alice Assinger, Anita Pirabe, Jonas Santol, Abbas Muhammad, David Kuroki-Hasenöhrl

**Affiliations:** 1 Ludwig Boltzmann Institute for Cardiovascular Research, Vienna, Austria; 2 Institute of Vascular Biology and Thrombosis Research, Center for Physiology and Pharmacology, Medical University of Vienna, Vienna, Austria; 3 Croma Pharma GmbH, Leobendorf, Austria

**Keywords:** fluid-platelet rich fibrin, performance, platelet-rich plasma, platelets, rejuvenation

## Abstract

**Objective:**

This study aimed to validate and characterize the efficacy of a previously developed anticoagulant-free, single soft-spin centrifugation device for the preparation of autologous fluid-Platelet-Rich Fibrin (f-PRF).

**Introduction:**

f-PRF is generated via one-step soft-spin centrifugation of all blood to produce a platelet-enriched plasma for regenerative medicine use, without the need for additional chemical agents. This study validated the performance of a novel single soft-spin f-PRF preparation system.

**Methods:**

Sixteen healthy volunteers (94% female, ages 23-52) donated blood for a comparative analysis between Exprecell™ and Arthrex ACP^®^ Double-Syringe systems. f-PRF was prepared using standardized centrifugation (420*xg*, 5 min) and characterized for cellular composition, platelet function, growth factors, and extracellular vesicles (EVs). Platelet activation was assessed via P-selectin expression and GPIIb/IIIa activation following stimulation using flow cytometry.

**Results:**

Exprecell™ yielded 20% more f-PRF volume (6.5–10.5 vs. 5.5–9.0 mL) with excellent cellular depletion (>99% erythrocyte, >95% leukocyte reduction). Platelet counts and function were similar between systems, with preserved *in vitro* agonist responses in terms of P-selectin expression and GPIIb/IIIa activation. Most growth factors remained below detection limits, and those detectable showed no differences between the devices. EV profiles from different cell types were also comparable.

**Conclusion:**

These findings support the Exprecell™ single soft-spin methodology, demonstrating that anticoagulant-free f-PRF preparation achieves functional equivalence to conventional methods while providing a statistically significant increase in volume yield and procedural simplicity. The closed system design reduces contamination risk and Luer-lock compatibility facilitates integration into clinical workflows. Maintained *in vitro* biological activity supports clinical utility for this innovative point-of-care f-PRF preparation device. Future studies are needed to demonstrate the clinical benefit of the f-PRF obtained.

## 1 Introduction

Autologous platelet-rich plasma (PRP) and fluid-Platelet-Rich-Fibrin (f-PRF) can be derived from autologous whole blood as a concentrated plasma fraction containing supraphysiological platelet levels and their associated growth factors. These bioactive mediators include transforming growth factor-β (TGF-β), platelet-derived growth factor (PDGF), epidermal growth factor (EGF), vascular endothelial growth factor (VEGF), insulin-like growth factor-1 (IGF-1), fibroblast growth factor (FGF) and serotonin (5-HT), which collectively orchestrate tissue regeneration and healing processes (Trull-Ahuir et al., 2020).

Autologous PRP and f-PRF have emerged as versatile regenerative therapies across multiple medical specialties. Clinical evidence supports its efficacy in orthopedic applications, including osteoarthritis management (Kemmochi, 2025; Xiong et al., 2023; Yoshioka et al., 2024), periodontal therapy (Niemczyk et al., 2024; Rattanawonsakul et al., 2025), peripheral neuropathy treatment (Malahias et al., 2018; Sánchez et al., 2014), dermatologic conditions such as acne scarring (Gaumond et al., 2024; Ahramiyanpour et al., 2025), androgenetic alopecia (Anitua et al., 2025; Ghanem et al., 2025; Nguyen and Nguyen, 2025), pigmentary disorders (Cecerska-Heryć et al., 2022; Feng et al., 2025), and chronic wound healing impairments (Huber et al., 2021; Ullah et al., 2022). In aesthetic medicine, PRP and f-PRF are known to improve skin quality, texture, and tone, either as stand-alone treatments (Tsai et al., 2024; Gorodilova et al., 2024); or in combination with laser therapy (Wu et al., 2022) or microneedling (Diab et al., 2023; Vesala and Nacopoulos, 2025). Multiple randomized controlled trials, systematic reviews, and meta-analyses demonstrate that PRP/f-PRF therapy enhances tissue repair and improves patient outcomes while maintaining an excellent safety profile (Xiong et al., 2023; Anitua et al., 2025; Ghanem et al., 2025; Nguyen and Nguyen, 2025; Feng et al., 2025; Biben et al., 2025).

The final composition of PRP and f-PRF depends critically on the processing methods, with protocol variations yielding products that differ significantly in biological properties and therapeutic potential (Ghanem et al., 2025; Degen et al., 2017). The preparation process involves three fundamental steps: blood collection, centrifugation-based separation, and platelet concentrate isolation. Different commercial devices employ varying protocols - including differences in gravitational force, duration, and centrifugation cycles, that substantially affect platelet concentration, leukocyte content, and overall cellular composition (Ghanem et al., 2025; Muthu et al., 2022; Magalon et al., 2021; Du et al., 2018). Furthermore, traditional PRP preparations often require anticoagulants, which introduce additional clinical concerns. Anticoagulants such as acid-citrate-dextrose (ACD) and sodium citrate (SC) can potentially interfere with the regenerative and pro-angiogenic properties of PRP: citrate chelation of Ca^2+^ in SC; and the presence of dextrose in the case of ACD, can alter platelet activation dynamics and endothelial responses, with measurable consequences for tissue repair (Aizaw et al., 2020; do Amaral et al., 2016; Ulasli et al., 2022). The use of anticoagulants in such preparations has been associated with local adverse reactions, including allergy (Latalski et al., 2019), delayed tissue healing and inflammatory intradermal papule formation following injection (Oneto et al., 2020). A study comparing patient-related discomfort in PRP injections for facial rejuvenation and hair loss using two different anticoagulants (ACD-A, SC), showed that both anticoagulants affected patients’ pain perceptions during the injections (Görgü et al., 2020; Karanfil et al., 2024). Additionally, in a retrospective study of 225 patients with knee osteoarthritis, researchers investigated the relationship between anticoagulant use in the preparation of PRP and post-treatment pain (Chen et al., 2024). Patients were categorized based on the type and amount of anticoagulant used during PRP preparation (4% SC 0.6 mL, 4% SC 1 mL, 4% SC 2 mL, heparin 0.1 mL, and heparin 0.2 mL) (Chen et al., 2024). Patients in the 4% SC 0.6 mL and heparin 0.1 mL groups experienced less pain after PRP treatment than did patients in the high-dose anticoagulant group, and the joint fluid of patients with pain in these groups had lower levels of inflammatory markers (Chen et al., 2024). Moreover, safety data from transfusion and apheresis practice further emphasize that citrate exposure can induce symptomatic hypocalcemia due to ionized calcium depletion, and regulatory labeling explicitly restricts 4% sodium citrate to extracorporeal anticoagulation rather than direct tissue infusion, underscoring biocompatibility concerns (Navkudkar et al., 2021). Together, these mechanistic, translational, and human clinical findings provide a compelling rationale for developing anticoagulant-free PRP systems, such as fibrin-based concentrates, which avoid citrate exposure and have demonstrated promising regenerative potential across multiple clinical fields.

To address these limitations, point-of-care (POC) devices based on a single “soft-spin” methodology have been developed. This approach employs relatively low centrifugal forces to separate whole blood into a dense erythrocyte layer and a f-PRF supernatant, suitable for immediate reinjection within 30 min (Gulliksson, 2012; Ratajczak et al., 2018). The result is a cost-effective f-PRF containing microaggregates that results in a gel-like formulation upon reinjection (Rattanawonsakul et al., 2025; Wang X. et al., 2019; Alsabri et al., 2024), forming a 3D network of platelet rich fibrin that captures growth factors (GFs) (Ghanaati et al., 2014), and providing a slow continuous release of GFs for up to 10-day post-reinjection, thereby producing longer-lasting effects compared to conventional PRP (Rattanawonsakul et al., 2025; Kobayashi et al., 2016).

**FIGURE 1:**
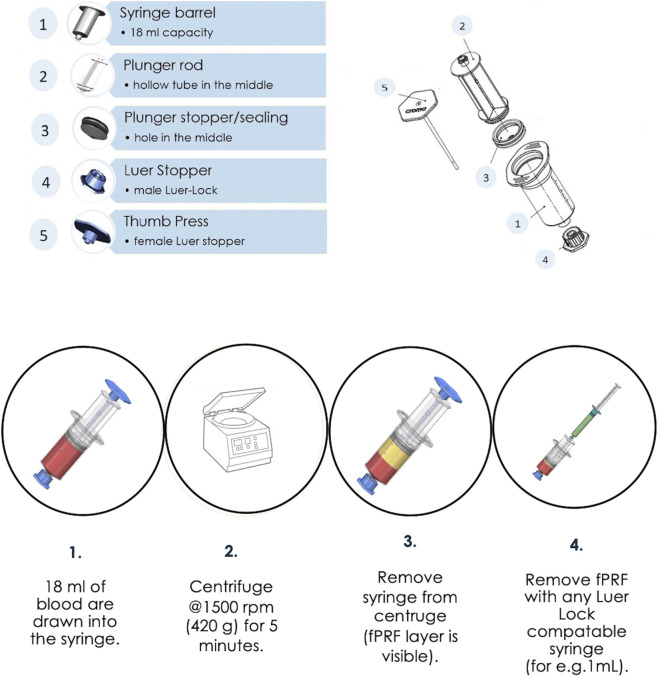
Schematic representation of the components (top), and the single soft-spin method for the preparation of fluid-Platelet Rich Fibrin (f-PRF) without additives using Exprecell (bottom).

Building on this concept, a closed-system device, Exprecell™ was developed for autologous f-PRF preparation using single soft-spin protocol without anticoagulation. The device is CE certified according to the European Medical Device Regulation, with market authorization held by Croma Pharma GmbH (Leobendorf, Austria). As illustrated in Figure 1, the device comprises a syringe barrel (18 mL capacity), plunger rod with an integrated f-PRF withdrawal/transfer channel that enables controlled aspiration up to a predefined maximum volume, a plunger stopper, a closure stopper, and thumb press with occluding mechanism which prevents backflow of blood into the f-PRF channel during collection.

The aim of this validation study was to comprehensively characterize the performance of this previously developed f-PRF preparation system, including cellular composition, growth factors and inflammatory mediator profiles, and *in vitro* functional properties, in comparison with the established Arthrex ACP^®^ Double-Syringe System (Arthrex GmbH, Munich, Germany).

## 2 Materials and methods

### 2.1 Blood sample collection

In this pre-clinical validation study, 16 healthy adult volunteers (94% females, 6% males, age range: 23–52 years old) donated blood following informed consent. The study was approved by the Ethics Committee of the Medical University of Vienna (ECS 2111/2024), and all relevant national and international codes of practice were respected (i.e., Declaration of Helsinki, Conventions of the Council of Europe on Human Rights and Biomedicine, Good Scientific Practice (GSP) Guidelines). All analysis were made at the Institute of Vascular Biology and Thrombosis, Center of Physiology and Pharmacology, Medical University of Vienna, Austria. All blood donors met strict health criteria: free of medication, neither pregnant nor breastfeeding, no known systemic diseases, normal body temperature, and no blood count abnormalities at the time of blood sample collection. In total, 33 mL of blood were collected per donor using a venipuncture blood collection system following sterile aseptic technique (21Gx3/4″ butterfly needle, Vacuette™, Greiner Bio-One GmbH, Kremsmünster, Austria), in combination with the following f-PRF collection systems: Arthrex ACP^®^ Double-Syringe System (15 mL, Arthrex GmbH, Munich, Germany), and Exprecell™ (18 mL, Croma Pharma GmbH, Leobendorf, Austria). Blood drawing was preformed from left arm (Exprecell™) and right arm (Arthrex ACP^®^) of the same donor, or *vice versa*.

### 2.2 Whole blood analysis

Prior to centrifugation, 50 μL aliquots of whole blood were collected from each f-PRF system for comprehensive hematological analysis using standardized automated analyzers (Horiba ABX Micros 60, Horiba Medical, Kyoto, Japan). Complete blood count parameters, including platelet count, leukocyte count, erythrocyte count, and hematocrit percentage, were measured for each sample (n = 16). Anticoagulants were added only to blood aliquots to permit analyzer readings and prevent clogging of the instruments.

### 2.3 Preparation of f-PRF

Within 5 min of venipuncture, blood samples were centrifuged at 420xg for 5 min under standardized conditions (Horizon 24-AH, Drucker Diagnostics, Port Matilda, PA, United States setting A, equivalent to 1500 rpm). Post-centrifugation, distinct layer separation was observed, with erythrocyte forming the lower layer and f-PRF the superior layer. For f-PRF extraction, blood-filled collection devices were secured within their positioning blisters. In the Exprecell™ system, the thumb press was aseptically removed from the distal plunger end to expose the Luer-lock interface. A sterile, Luer-lock syringe (e.g., B. Braun GmbH, Melsungen, Germany) was attached to the plunger rod of Exprecell™, enabling controlled f-PRF withdrawal through the designated channel up to the maximum mark. Gentle retraction of the syringe plunger generated a negative pressure within the Exprecell™ device, facilitating displacement and transfer of f-PRF. In the Arthrex ACP^®^ system, f-PRF was aspirated using the integrated secondary syringe provided in the kit, following identical aseptic handling procedures.

### 2.4 F-PRF characterization and analysis

Following f-PRF extraction, all samples (n = 16) underwent comprehensive characterization using standardized analytical protocols.

*Coagulation and volume assessment*: Clotting kinetics were assessed by monitoring the time-to-clot formation for each collection device and the total f-PRF volume obtained from each system was quantified.

*Platelet concentration and recovery*: Platelet enrichment achieved during centrifugation and was calculated using the Platelet Concentration Factor (PCF):

$$Platelet\;concetration\;factor\;\left(PCF\right)=\frac{Platelet\;concentrate\;\left(PC\right)}{Baseline\;\left(BL\right)}$$

Platelet recovery was calculated as Platelet Yield (PY):

$$Platelet\;yield\;\left(PY\right)=\frac{PC\times Volume\;PC\;\left(VPC\right)}{BL\times Volume\;of\;the\;sample\;processed\;\left(VBL\right)}$$

*Physicochemical properties*: The pH of each platelet concentrate was measured using a calibrated micro-electrode system (Seven Compact pH meter S210, Mettler Toledo, Colombus, OH, United States) to assess physiological compatibility.

### 2.5 Growth factors and inflammatory mediator analysis

*Sample activation and processing*: A subset of f-PRF samples (n = 12) underwent controlled platelet activation via shear stress (high speed centrifugation: 1000×g for 2 min) to induce degranulation and growth factor release. Supernatants were collected for biomarker analysis using a LEGENDplex bead-based multiplex immunoassay (BioLegend) coupled with flow cytometric analysis (CytoFLEX, Beckman Coulter, Brea, CA, United States), with selected analytes validated by enzyme-linked immunosorbent assay (ELISA) (Quantikine ELISA kits, R&D Systems, Minneapolis, MN, United States).

*Growth factor panel*: Analyzed growth factors included: Platelet-derived growth factor isoforms (PDGF-AA and PDGF-BB), transforming growth factor-alpha (TGF-α), vascular endothelial growth factor (VEGF), basic fibroblast growth factor (bFGF), angiopoietin-2, erythropoietin, hepatocyte growth factor (HGF), epidermal growth factor (EGF), granulocyte-colony stimulating factor (G-CSF), macrophage-colony stimulating factor (M-CSF), granulocyte-macrophage colony-stimulating factor (GM-CSF), stem cell factor (SCF), insulin-like growth factor-1 (IGF-1), and transforming growth factor-beta (TGF-β1).

*Inflammatory Mediator Panel*: Analyzed growth factors included: interleukins IL-1β, IL-2, IL-4, IL-6, IL-8, IL-10, IL-17A, and IL-12p70; chemokines CXCL10 (IP-10) and CCL2 (MCP-1); tumor necrosis factor-alpha (TNF-α); and interferon-gamma (IFN-γ).

### 2.6 Functional analysis

Platelet responsiveness in f-PRF was assessed using dose-response stimulation assays with 3, 10 and 20 μM adenosine diphosphate (ADP, Sigma). Platelet degranulation was quantified via surface expression of P-selectin (mouse anti-human CD62P-Brilliant Violet 605,1:100, clone AK4; BioLegend), and platelet aggregation was evaluated through glycoprotein IIb/IIIa (GPIIb/IIIa) activation using PAC-1 monoclonal antibody binding (FITC mouse anti-human PAC-1, 1:60, BD Bioscience). After 20 min of staining, platelets were fixed with 1% formaldehyde and analyzed on a CytoFLEX flow cytometer using CytExpert 2.4 software (both Beckman Coulter). The percentage of P-selectin-positive and PAC-1-positive platelets following agonist stimulation served as quantitative indicators of platelet responsiveness after processing.

### 2.7 Extracellular vesicle (EV) analysis

EVs were analyzed in platelet-free plasma prepared by sequential centrifugation to eliminate residual platelets and prevent artifactual signals as described previously (Mussbacher et al., 2017). EVs were gated within defined size ranges using calibrated fluorescent beads as reference standards (100–800 nm, Nanobead Calibration Kit, Bangs Laboratories).

*EV identification and phenotyping:* EVs were identified via lactadherin binding (bovine lactadherine Alexa Fluor-647, 1:80, Cell Systems), with phalloidin (Phalloidin-iFluor 488, Abcam) staining used to exclude debris. Cellular origin of EVs was determined using specific surface markers: glycoprotein IIIa (mouse anti human CD61-FITC, clone: VIPL2, Biolegend) for platelet-derived EVs, pan-leukocyte antigen (mouse anti human CD45 Brilliant Violet-605, clone: HI30, Biolegend) for leukocyte-derived EVs, vascular endothelial cadherin (mouse anti human CD144 PE/Cyanine7, clone:BV9, Biolegend) for endothelial cell-derived EVs and glycophorin A (recombinant anti human CD235a PerCP/Cyanine5.5 a, Biolegend) for erythrocyte-derived EVs. All monoclonal antibodies underwent pre-centrifugation at 20,000×g for 2 min to remove protein aggregates. Buffers were sterile filtered through 0.1 μm membranes prior to use. Daily instrument standardization was performed using fluorescent calibration beads of defined sizes (0.1, 0.2, 0.5, and 0.8 μm) to ensure measurement accuracy and reproducibility. EV detection and characterization were performed using multiparameter flow cytometry (CytoFLEX, Beckman Coulter, Brea, CA, United States).

### 2.8 Statistical analysis

Baseline comparisons of continuous variables between the Arthrex ACP^®^ and the Exprecell™ were performed using unpaired Student's t-tests, while categorical variables were analyzed using chi-square tests for independence.

*Platelet function analysis, data distribution and transformation*: The effects of different agonist concentrations on platelet function parameters were evaluated using Analysis of Covariance (ANCOVA) models, with corresponding baseline values included as covariates to control for inter-individual variability.

*Software and Statistical Significance:* All statistical analyses were performed using SPSS version 23.0 (IBM Corporation, Armonk, NY, United States) and GraphPad Prism version 6.0 (GraphPad Software, San Diego, CA, United States). Statistical significance was defined as p < 0.05 for all comparisons.

## 3 Results

**FIGURE 2:**
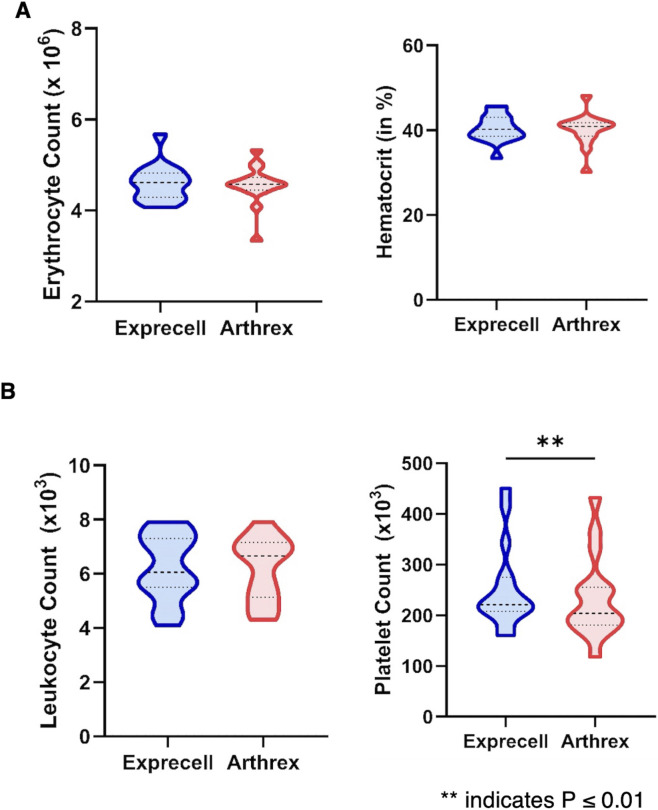
Blood counts in whole blood from the same donor collected using either the Exprecell or Arthrex ACP device: **(A)** Erythrocyte count and hematocrit were comparable between devices, **(B)** Leukocyte counts are similar in Exprecell and Arthrex, while platelet counts are significantly higher in Exprecell; data are shown as n = 16; ** indicates P ≤ 0.01.

Blood count analysis was performed on whole blood samples prior to f-PRF processing, with quantification of leukocyte count, erythrocyte count, platelet count, and hematocrit percentage per milliliter. Descriptive statistics for these baseline hematological parameters are presented in Figure 2.

While erythrocyte counts (Figure 2A), hematocrit and leukocyte counts (Figure 2B) were comparable between the two preparation systems, statistical analysis revealed a significant difference in baseline platelet counts between the two f-PRF preparation systems (Figure 2B).

Whole blood collected with the Exprecell™ system demonstrated a mean platelet count of 234 ± 78 × 10^3^/μL compared to 211 ± 69 × 10^3^/μL for the Arthrex ACP^®^ Double-Syringe system (95% confidence interval: −37.5 to −7.7 × 10^3^/μL; p = 0.0063). The Exprecell™ device permits extraction of up to 18 mL of blood volume, whereas the Arthrex ACP^®^ Double-Syringe system allows recovery of 15 mL total volume. Following standardized centrifugation (420*xg* for 5 min), whole blood samples consistently separated into distinct phases: inferior layer - i.e., densely packed erythrocytes forming the red blood cell pellet; and superior layer - i.e., f-PRF, with its characteristic yellow coloration.

**FIGURE 3:**
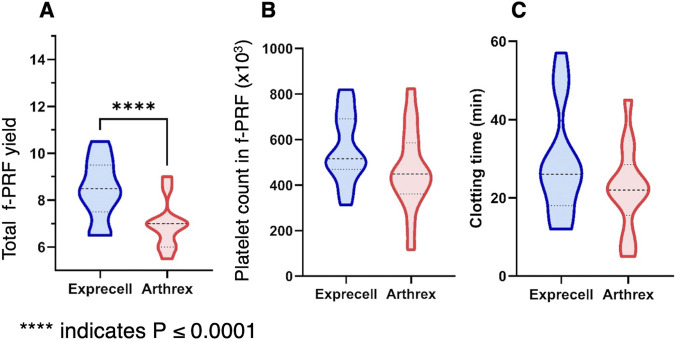
Comparison of f-PRF parameters between Exprecell and Arthrex ACP systems: **(A)** Total volume of fluid-Platelet-rich Fibrin (f-PRF) obtained from the two devices; **(B)** Platelet count in f-PRF; **(C)** Clotting time of f-PRF generated by both systems. Data are shown as n = 16; **** indicates P ≤ 0.0001.

Comparative analysis demonstrated measurable differences in f-PRF yield between the two preparation systems (Figure 3A). The Exprecell™ device produced f-PRF volumes ranging from 6.5 to 10.5 mL (mean 8.45 ± 1.3 mL), while the Arthrex ACP^®^ system yielded 5.5–9.0 mL (mean 6.95 ± 1.0 mL). This volumetric difference corresponds directly with the initial blood draw capacity, as the Exprecell™ processes 18 mL of whole blood compared to 15 mL for the Arthrex ACP^®^, resulting in approximately 20% greater f-PRF yield with the Exprecell™ system. Despite the volumetric differences, platelet concentrations within the recovered f-PRF showed no statistically significant differences between the two systems (Figure 3B, p > 0.05).

Clotting time varied considerably in both systems, ranging from 12 to 57 min for Exprecell™ and 5–45 min for Arthrex ACP^®^. Mean clotting time for the Exprecell™ was 29.15 ± 14.22 min, while Arthrex ACP^®^ exhibited a mean clotting time of 22.38 ± 11.0 min, with no statistically significant differences being observed (Figure 3C, p = 0.161). These slightly faster clotting events in the Arthrex device might contribute to the lower platelet counts observed in whole blood.

**FIGURE 4:**
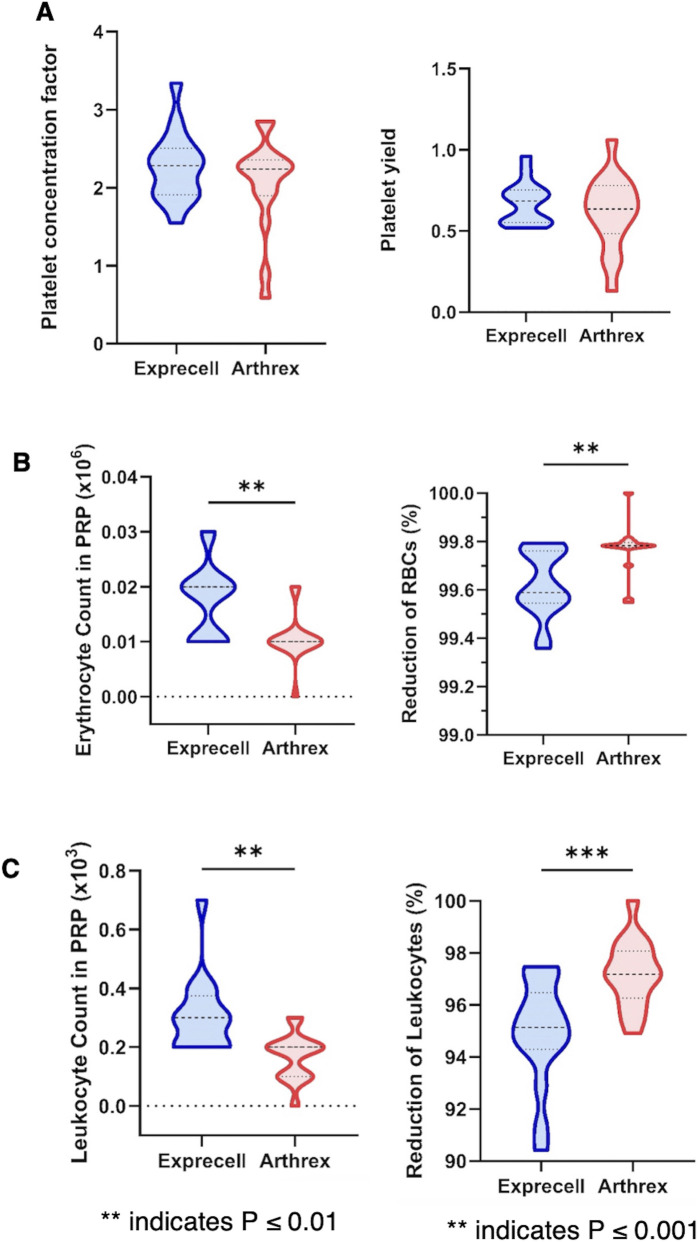
Platelet, erythrocyte, and leukocyte composition in f-PRF from Exprecell and Arthrex ACP systems: **(A)** Platelet concentration factor (left) and Platelet yield in f-PRF obtained from the two devices (right); **(B)** Erythrocyte counts (left) and Reduction in f-PRF for both systems (right); **(C)** Leukocyte counts (left) and reduction in f-PRF for both systems (right). Data are shown as n = 16; ** indicates P ≤ 0.01; ***indicates P ≤ 0.001.

The platelet concentration factor, with 2.3 ± 0.3 in Exprecell™ vs. 2.1 ± 0.6 in Arthrex ACP^®^, showed no statistically significant differences between systems (Figure 4) and platelet yield was statistically equivalent between the two preparation systems (Exprecell™: 67.2% ± 16.0% vs. Arthrex ACP^®^: 61.5% ± 16.6%). Both devices demonstrated excellent erythrocyte reduction, achieving >99% depletion compared to whole blood baseline values, though Exprecell™ showed significantly higher residual erythrocytes (p < 0.01; Figure 4B). Leukocyte reduction was similarly effective, with the Exprecell™ system achieving approximately 95% reduction and the Arthrex ACP^®^ demonstrating approximately 97% reduction relative to baseline whole blood counts (p < 0.01; Figure 4C).

**FIGURE 5:**
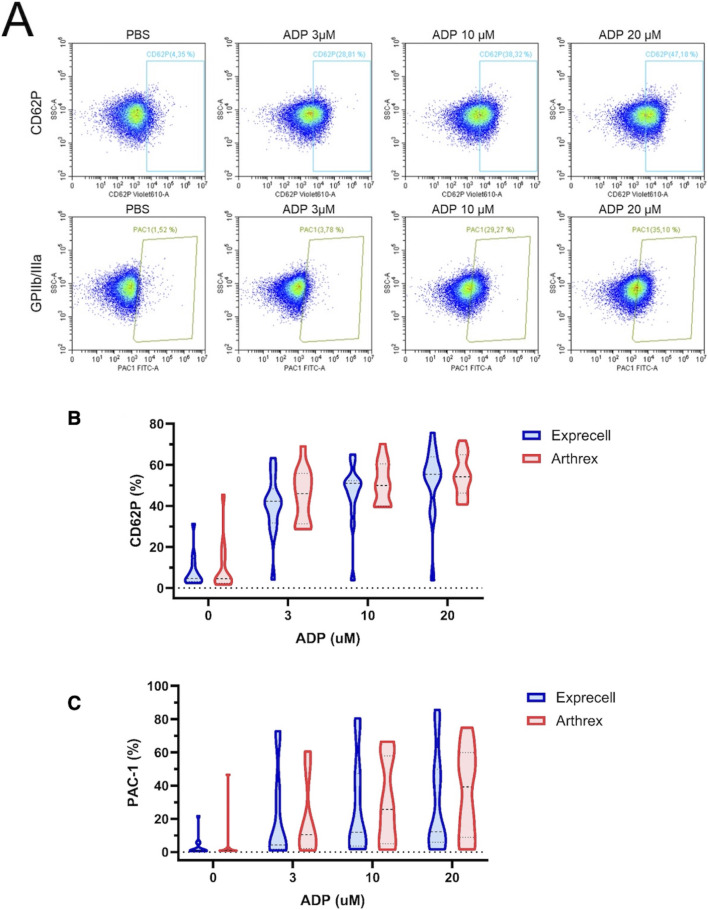
Platelet activation in response to adenosine diphosphate (ADP) in platelet-rich plasma from Exprecell and Arthrex ACP systems: **(A)** Scatter plots of platelets showing surface expression of P-selectin (CD62P) and activation of GPIIb/IIIa in response to different concentrations of ADP (3–20 µM), representative images; **(B)** Quantification of CD62P expression; **(C)** Quantification of GPIIb/IIIa activation. Data are shown as n = 16.

Next, we analyzed platelet function in response to different concentrations of ADP in both systems. Blood from both systems demonstrated a concentration-dependent increase in P-selectin surface expression and GPIIb/IIIa activation upon ADP stimulation (Figures 5A–C). These response pattern indicate that platelets efficiently released granule contents to comparable extents, with no statistically significant differences observed between the two devices (p > 0.05).

**FIGURE 6:**
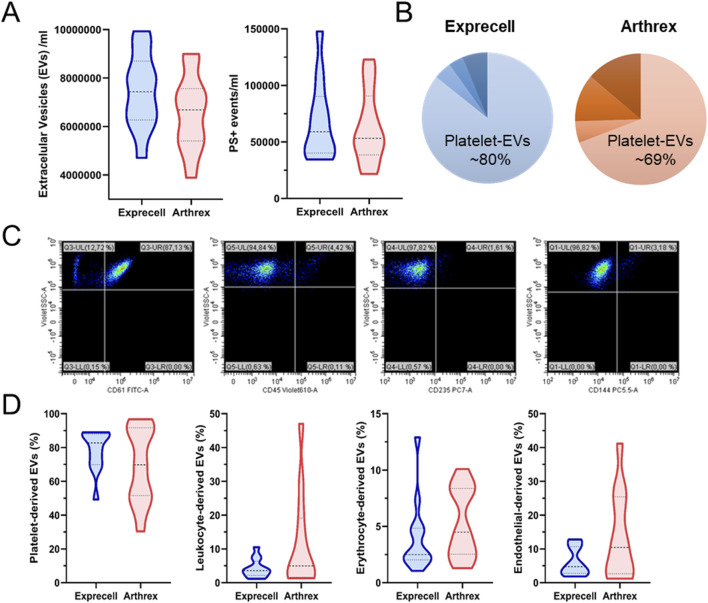
Extracellular vesicles (EVs) in platelet-free plasma from Exprecell and Arthrex ACP double syringe systems: **(A)** Total EVs and phosphatidylserine (PS)-positive EVs measured in platelet-free plasma; **(B)** Pie charts showing the cellular origin of EVs (%); colors from light to dark represent platelets, endothelial cells, leukocytes, and erythrocytes; **(C)** Representative images of EVs and their cellular origin; **(D)** Quantification of EVs and their cellular origin (n = 16), detected via lactadherin positivity combined with cell-specific antibodies (CD61, CD45, CD235a, CD144).

Since EVs are major carriers of bioactive substances, we analyzed the concentration and cellular origin of EVs present in f-PRF. All EV subpopulations displayed comparable distribution patterns across the two f-PRF preparation systems, with no statistically significant differences observed between the Exprecell™ and Arthrex ACP^®^ systems (p > 0.05, Figure 6A). Phenotypic characterization revealed that EVs obtained using Exprecell™ comprised 80% platelet-derived vesicles, compared to 69% for Arthrex ACP^®^. However, these differences did not reach statistical significance (Figures 6B–D).

**FIGURE 7:**
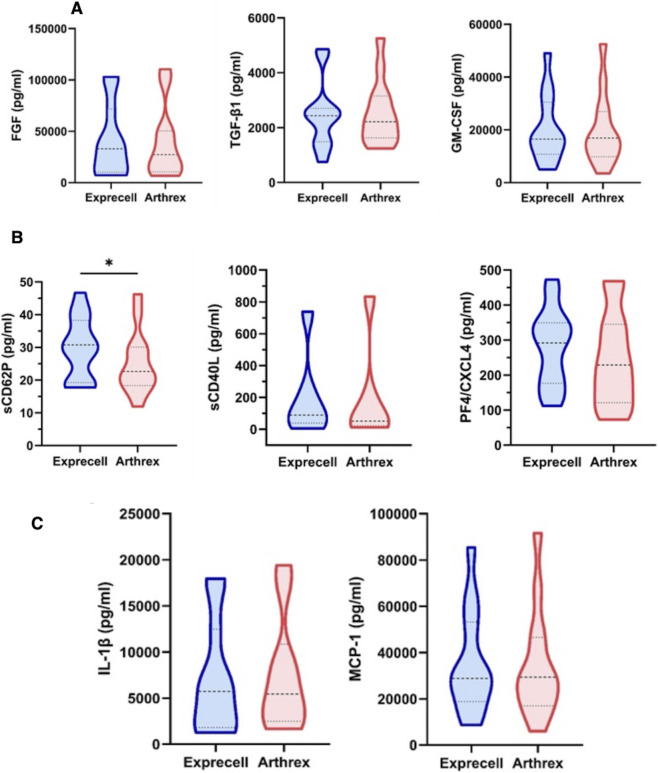
Growth factor, platelet-derived mediator, and inflammatory cytokine profiles in f-PRF supernatants. **(A)** Quantification of basic fibroblast growth factor (bFGF), granulocyte-macrophage colony-stimulating factor (GM-CSF), and transforming growth factor-beta 1 (TGF-β1); **(B)** Quantification of soluble P-selectin (sCD62P), soluble CD40L (sCD40L), and platelet factor 4 (PF4/CXCL4); **(C)** Quantification of interleukin-1β (IL-1β) and CCL2 (MCP-1) release in the supernatant of f-PRF derived from the Exprecell and Arthrex ACP systems. Data are shown as n = 16; * indicates p < 0.05.

To further characterize the bioactive components of f-PRF preparations, we analyzed growth factor release profiles following shear stress activation. While a comprehensive panel of growth factors was assessed in f-PRF-derived supernatants from both preparation systems, most analytes were below the detection limits of the respective assays. Three growth factors were consistently measurable above threshold levels: basic fibroblast growth factor (bFGF), granulocyte-macrophage colony-stimulating factor (GM-CSF), and transforming growth factor-beta 1 (TGF-β1) (Figure 7A). Quantitative analysis revealed comparable concentrations of these growth factors between the two systems, with no statistically significant differences observed (p > 0.05). Platelet-specific soluble mediators, including soluble P-selectin (sP-selectin), soluble CD40L (sCD40L), and platelet factor 4 (PF4/CXCL4), were also quantified (Figure 7B). While CD40L and PF4/CXCL4 concentrations were similar across systems, sP-selectin levels were significantly higher in Exprecell™-derived supernatants. Inflammatory mediator profiling was conducted on the same supernatant samples used for growth factor analysis. Consistent with previous findings, most cytokines were below detection thresholds. Among the detectable analytes, interleukin-1β (IL-1β) and CCL2 (MCP-1) showed no significant differences between the two systems (Figure 7C).

## 4 Discussion

This validation study demonstrated that the novel Exprecell™ system reliably produces clinically viable f-PRF with *in vitro* preserved regenerative and aesthetic potential, establishing non-inferiority to the established Arthrex ACP^®^ Double-Syringe system across multiple analytical and *in vitro* functional parameters. Despite notable technical design differences, both devices generated functionally equivalent f-PRF suitable for therapeutic and aesthetic applications (Table 1).

**TABLE 1:** Summary table comparing performance metrics between systems. EV: Extracellular Vesicles. SD: Standard Deviation.

Performance parameters	Exprecell^TM^	Arthrex ACP® double-syringe systems
Blood collection capacity	18 mL	15 mL
f-PRF volume obtained	6.5–10.5 mL (mean: 8.45 ± 1.3 mL) **** P ≤ 0.0001 (∼ 20% higher yield)	5.5 - 9.0 mL (mean: 6.95 ± 1.0 mL)
Performance	- Leukocyte reduction: 95% - Erythrocyte reduction: > 99% vs whole blood baseline values - Platelet concentration factor: 2.3 ± 0.3 - EVs per mL (Mean ± SD): 745 0238 ± 156 3853 - EV origin: 80% platelet derived	- Leukocyte reduction: 97% - Erythrocyte reduction: > 99% vs whole blood baseline values - Platelet concentration factor: 2.1 ± 0.6 - EVs per mL (Mean ± SD): 613 9494 ± 232 2516 - EV origin: 69% platelet derived
Functional equivalence	- Platelet function: Preserved and comparable agonist responses - Growth factors: Comparable levels (bFGF, GM-CSF, TGF-β1) - Inflammatory mediators: Similar profiles - EV populations: Comparable distribution	
Implications	Both systems produce functionally equivalent f-PRF suitable for regenerative and aesthetic applications, with: - Excellent cellular depletion - Preserved platelet activation capacity - Comparable biological activity - Similar growth factor and EV profiles Note: Most growth factors (PDGF-AA, PDGF-BB, TGF-α, VEGF, EGF, HGF, and others) remained below detection limits in both systems. Only bFGF, GM-CSF, and TGF-β1 were consistently detectable.	

A key advantage of Exprecell™ was its superior volume yield, producing approximately 20% more f-PRF (6.5–10.5 mL vs. 5.5–9.0 mL) due to larger processing capacity (18 mL vs. 15 mL). Importantly, this increased volume did not compromise platelet quality, as both systems achieved comparable platelet concentrations, growth factor profiles, and *in vitro* functional properties. Clinically, this higher yield may reduce the number of blood draws required per patient, improving procedural efficiency for applications requiring larger or multiple injection volumes.

Mean clotting time for the Exprecell™ was 29.15 ± 14.22 min, while Arthrex ACP^®^ exhibited a mean clotting time of 22.38 ± 11.0 min, with no statistically significant difference. The slight longer clotting time observed with Exprecell indicates a potential procedural advantage, as the f-PRF remains in a fluid state for an extended period. This provides healthcare professionals with greater working time for handling and applying fluid-PRF in clinical practice.

Both devices achieved excellent erythrocyte (>99%) and leukocyte (>95%) depletion, consistent with previous reports, showing that single soft-spin centrifugation promotes platelet-enrichment while maintaining product integrity (Ghanem et al., 2025; Perez et al., 2014; Pochini et al., 2016).

*In vitro* functional analyses confirmed that both systems preserved essential platelet activities. Elevated PF4/CXCL4 concentrations confirmed successful platelet activation and degranulation following shear stress activation with concentrations far exceeding physiological plasma concentrations (4–24 ng/L), consistent with previously published activation release dynamics (Chesterman et al., 1978). Conversely, soluble CD40L levels remained within baseline ranges, likely reflecting the short post-processing interval prior to measurement, as CD40L release involves delayed shedding of its soluble form (Kojok et al., 2018).

In contrast, soluble P-selectin (CD62P) levels were significantly elevated following shear stress activation in both systems, confirming α-granule degranulation and platelet activation (Katayama et al., 1993; Holmes et al., 1999). Platelet function analysis revealed that both systems yielded resting yet fully responsive platelets, as indicated by normal agonist-induced P-selectin expression and GPIIb/IIIa activation.

Growth factor analysis revealed no significant inter-system differences among detectable analysts (bFGF, GM-CSF, and TGF-β1) and inflammatory mediator profiles were similarly consistent, suggesting equivalent regenerative potential, which needs to be studied in a further clinical study.

EV characterization provided additional insights into f-PRF quality, with both systems producing similar overall EV populations derived from various cellular origins. Although Exprecell™ showed a trend toward higher platelet-derived EVs (80% vs. 69%), both preparations preserved physiologically relevant EV populations possibly associated with regenerative, hemostatic and inflammatory properties, although further studies are needed. The preservation of platelet function underscores the advantage of the single soft-spin anticoagulation-free methodology, which maintains platelet integrity and hemostatic capacity better than more aggressive centrifugation protocols (Ghanem et al., 2025; Nguyen and Nguyen, 2025; Muthu et al., 2022; Alsabri et al., 2024).

This approach also minimizes the risk of anticoagulant interference with physiological coagulation processes and mirrors real-world clinical workflow. Interestingly, the detection of FGF, GM-CSF, TGF-β1, IL-1β and CCL2 (MCP-1) indicate that platelets remained intact during f-PRF preparation with Exprecell™ as well as Arthrex ACP^®^, allowing gradual release of bioactive molecules post-application. In Exprecell™, the extended PRP withdrawal channel enables precise layer isolation while minimizing red blood cell contamination. Feedback from users emphasized high satisfaction with handling and usability. The preserved biological activity across all measured parameters supports the clinical utility of Exprecell™ as a reliable, efficient, and biocompatible f-PRF preparation system for point-of-care use.

Overall, these findings validate the performance of the Exprecell™ system and highlight its practical advantages in terms of greater f-PRF yield, simplified extraction and user-friendly design. To mimic a clinical setting, we have designed the study protocol where blood is drawn and centrifuged at 5 min (i.e., blood is kept in the device before transfer into the centrifuge machine). As such, this anticoagulant-free f-PRF preparation protocol requires immediate centrifugation following venipuncture to prevent clot formation. This workflow is most feasible in clinical settings where point-of-care centrifugation equipment is readily accessible within or adjacent to the procedure room, enabling processing within 5 min of blood collection to prevent clot formation. Suitable environments include dental and oral surgery offices, dermatology and aesthetic medicine clinics, orthopedic surgery centers, and sports medicine facilities - i.e., settings where dedicated procedural spaces allow for streamlined blood collection and processing. On the other hand, this approach may be less practical in high-volume outpatient clinics without dedicated point-of-care equipment, facilities requiring off-site laboratory processing, or settings where procedural, logistical, or staffing constraints bar immediate centrifugation. Successful implementation of the Exprecell™ system requires pre-procedure equipment setup; and, ideally, a team-based workflow coordinating phlebotomy and centrifugation to minimize processing delays.

This study has limitations. First, this study did not systematically evaluate the tolerance of f-PRF quality to delays between venipuncture and centrifugation - we specifically noted time from blood draw to transferring the blood filled Exprecell/Arthrex device in the centrifuge and kept it 5 min for each device. As such, the acceptable time window before clot initiation compromises product quality and warrants further investigation to better establish workflow flexibility in diverse clinical settings.

Second, the sample size (n = 16) may limit statistical power for some parameters, and clinical efficacy was not assessed. Third, our study cohort had a strong gender imbalance (94% female), which can be acceptable for a pre-clinical validation study, but limits the generalizability of our findings. Future studies should be complemented with a more diversified patient population applied to different medical purposes (e.g., orthopedics, sports medicine), since it is known that sex-related differences in platelet biology are important in the context of the regenerative properties of PRP injections due to differences in the concentration and profile of growth factors and cytokines, as well as platelet activation and aggregation potential between males and females (Xiong et al., 2018). For example, male PRP contains higher levels of key growth factors such as FGF-b, PDGF-BB, and TGF-β1, as well as higher concentrations of both pro-inflammatory (IL-1β, TNF-α) and anti-inflammatory (IL-1 receptor antagonist) cytokines compared to female PRP (Xiong et al., 2018). These compositional differences may influence the regenerative efficacy of PRP, with male-derived PRP potentially providing a more robust stimulus for tissue repair and angiogenesis. Furthermore, female platelets exhibit greater aggregation and activation in response to stimulation; and estradiol enhances platelet activation in both sexes, suggesting that hormonal status modulates platelet function and may impact PRP performance in tissue regeneration (Coleman et al., 2019). Additionally, post-menopausal status in women is associated with changes in leukocyte composition and platelet function, which may further affect PRP quality and regenerative outcomes (Trevissón et al., 2022).

Fourth, relative to the differences seen in baseline platelet counts between the two systems, it is known that biological variation, including circadian rhythms, local hemodynamics, and microvascular differences, can influence platelet counts even within the same individual (Peng et al., 2004). As such, although we tried our best for comparable conditions and within the same post-collection time frame, it is known that slight differences in venous stasis, tourniquet application time, and the order of draw can affect platelet concentration by causing local hemoconcentration or platelet activation during sampling (Lancé et al., 2013). These conjecture of facts might have contributed to the difference observed in the baseline platelet counts seen between collection systems, although we believe it did not affect the downstream results of our study. Furthermore, since the blood draw was performed using Exprecell and Arthrex ACP from right and left arm of the same patient/donor, device related bias can be ignored as each donor serves as their own internal control. This paired-arm design reduces inter-individual variability and ensures that observed differences are attributable to the biological properties of the preparations rather than device-specific performance.

Fifth, the low growth factor detection rates likely reflect both the inherent biological characteristics of f-PRF and methodological constraints. The assays employed may lack the sensitivity required for PRF-based preparations in which growth factors are sequestered within a dense fibrin matrix rather than freely circulating at measurable levels, as is often the case in activated platelet-rich plasma. To mimic real-world clinical use of Exprecell, we deliberately did not use any anticoagulant in the processed samples analyzed by ELISA or FACS, thereby allowing physiological fibrin polymerization. Under these conditions, we believe most growth factors were either trapped within the fibrin matrix or released only at a later time points (e.g., around day 7), consistent with published observations by Zwittnig et al. (2022), and Wang Z. et al. (2019), who reported delayed and sustained release kinetics in fibrin-based preparations. As f-PRF is intended to provide gradual *in vivo* release rather than immediate burst kinetics, matrix-specific extraction protocols, more sensitive detection platforms, or modified activation strategies may be needed to better characterize its growth-factor profile and to establish correlations with clinical outcomes. This remains an important area for future research.

PRP/f-PRF evaluation in this study was restricted to healthy adult donors, as the biological activity and clinical efficacy of platelet-derived growth factors depend strongly on platelet functional quality, which is influenced by donor-related factors such as age, metabolic status, oxidative stress, and baseline platelet count (Costa et al., 2026; Rossi et al., 2023; Colomer-Selva et al., 2025). This variability is well-documented, with studies demonstrating that platelet functionality, growth-factor release, and final PRP composition differ substantially based on donor characteristics and comorbidities. To minimize these confounding variables during device-performance evaluation, we therefore used blood exclusively from healthy individuals and did not assess device behavior using diseased blood (Costa et al., 2026; Rossi et al., 2023; Colomer-Selva et al., 2025; Wu et al., 2025). This approach aligns with state-of-the-art evaluation principles for blood-contacting medical devices and hemocompatibility testing, which typically rely on standardized human blood or plasma from healthy donors to isolate device-related effects (ISO, 2017; FDA, 2024). Consequently, this is not considered a study limitation but an accepted and scientifically justified methodology for early-stage device characterization. Follow-up clinical studies should subsequently assess performance across relevant patient cohorts.

## 5 Conclusion

The findings demonstrate that the novel single soft-spin Exprecell™ device generates clinically viable f-PRF, while providing practical advantages in terms of higher yield and simplified processing. The demonstrated *in vitro* functional equivalence compared to an established high-quality system supports its use as a safe and effective alternative for f-PRF preparation, potentially reducing procedural complexity and minimizing risk of adverse reactions. Future randomized controlled trials comparing clinical outcomes are warranted to confirm and extend these laboratory-based findings.

## Data Availability

The raw data supporting the conclusions of this article will be made available by the authors, without undue reservation.
